# Progressive Ataxia and Cognitive Impairment Associated With SEZ6L2: Serum Neurofilament Light Chain as a Biomarker of Disease Activity

**DOI:** 10.1002/brb3.71900

**Published:** 2026-11-10

**Authors:** Carlo Fazio, Simone Regalbuto, Sebastiano Arceri, Davide Comolli, Alessandra Calculli, Giulio Trobbiani, Giuseppe Battaglia, Federica Zuliani, Matteo Gastaldi, Antonio Pisani

**Affiliations:** ^1^ Department of Brain and Behavioral Sciences University of Pavia Pavia Italy; ^2^ IRCCS Mondino Foundation Pavia Italy; ^3^ Department of Medicine, Surgery and Neuroscience University of Siena Siena Italy; ^4^ Neuroimmunology Research Section IRCCS Mondino Foundation Pavia Italy

**Keywords:** ataxia, autoimmune encephalitis, neurofilaments light chains, SEZ6L2

## Abstract

**Introduction:**

Autoimmune encephalitis (AE) associated with SEZ6L2 antibodies is a rare neurological disorder characterized by subacute onset of cerebellar ataxia, cognitive dysfunction, and extrapyramidal features. Few cases have been reported, and data on long‐term course and treatment response remain limited.

**Case Presentation:**

A 64‐year‐old woman developed progressive cerebellar ataxia, cognitive decline, and psychiatric symptoms starting insidiously after SARS‐CoV‐2 pneumonia in September 2022. Magnetic resonance imaging (MRI) and cerebrospinal fluid (CSF) analysis almost 1 year after onset were unremarkable. Due to progressive symptoms, she was admitted to our department in December 2024. Examination showed severe truncal and gait ataxia, memory impairment, and prominent frontal‐executive dysfunction. Brain MRI demonstrated bilateral hippocampal T2 hyperintensity with severe medial temporal lobe and cerebellar atrophy. Neuronal antibody testing with a tissue‐based assay (TBA) optimized for surface antigens showed strong neuropilar hippocampal and cerebellar staining in serum and CSF. A specific live cell‐based assay (CBA) confirmed that the reactivity could be attributed to SEZ6L2 antibodies. Plasmapheresis and high‐dose corticosteroids resulted in stabilization of gait impairment and mild cognitive improvement. Longitudinal plasma neurofilament light chain (NFL) monitoring showed initially high levels (85.12 pg/mL), followed by a drastic reduction some months after immunotherapy.

**Conclusions:**

AE can mimic neurodegenerative processes, and SEZ6L2 antibody testing should be considered in patients with progressive ataxia and limbic involvement, even when no additional clues point towards an autoimmune etiology. Notably, the condition manifested a partial response to immunotherapy, supported by NFL reduction. This suggests that NFL might represent a useful biomarker to monitor disease activity in these conditions.

## Graphical abstract

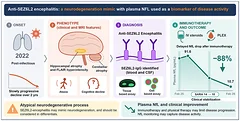

## 1 Introduction

Autoimmune encephalitides (AE) represent a heterogeneous group of neurological disorders often associated with antibodies against neuronal surface or intracellular antigens (Graus et al. 2016). Seizure‐related 6 homolog like 2 (SEZ6L2) AE is a recently recognized entity, first described in 2014 in a patient with subacute cerebellar ataxia and retinopathy (Yaguchi et al. 2014). SEZ6L2 is a neuronal surface protein prominently expressed in cerebellar cortex, basal ganglia, and hippocampal regions. In recent years, subsequent reports have expanded the phenotype to include parkinsonism, cognitive impairment, pyramidal signs, and mood disorders (Hoshina et al. 2026). As of mid‐2026, only 22 cases have been reported worldwide (not including ours), and critical gaps remain regarding clinical spectrum, disease course, and optimal treatment strategies.

We hereby present a 4‐year‐long case of SEZ6L2‐AE initially mimicking a primary neurodegenerative disorder. Scale for the Assessment and Rating of Ataxia (SARA) score was obtained at different timepoints. Treatment response and evolution were monitored using plasma neurofilament light chain (NFL) levels (Lumipulse NFL Plasma assay).

## 2 Case Presentation

A 64‐year‐old woman presented in December 2024 with a 2‐year history of progressive gait instability, falls, cognitive impairment, and depression. Her history was notable for breast ductal carcinoma in situ, surgically resected in March 2022. Symptoms began one month after hospitalization for SARS‐CoV‐2 pneumonia (late 2022) with mild unsteadiness. In the following months, the patient experienced mood changes and cognitive complaints that progressed insidiously over time. Neither the patient nor her relatives reported any acute phase or abrupt worsening at onset. Investigations performed about 1 year after onset, including cerebrospinal fluid (CSF) analysis with total tau, phosphorylated tau, and amyloid beta dosage, and neuropsychological testing, were largely unremarkable. Contrast‐enhanced magnetic resonance imaging (MRI) at that time (2023) showed only chronic small vessel disease, with periventricular leukoaraiosis and multiple, partly confluent gliotic areas in the white matter of both hemispheres (Fazekas Grade 2). No selective atrophy was described, as well as no pathological contrast enhancement. Incidental findings included a septated pineal cyst and an ectatic, tortuous basilar artery indenting the ventral pons.

Over the following months, she experienced progressive neurological deterioration with repeated falls and working difficulties due to attentional deficits, prompting referral to our center. On admission, examination revealed cerebellar dysarthria, truncal and gait ataxia with inability to perform tandem walking, and mild left‐sided limb ataxia (SARA Score 14). Neuropsychological assessment showed memory, visuo‐spatial, and executive dysfunction (MMSEc 25, clock drawing test [CDT] 4, frontal assessment battery [FAB] 10). Notably, brain MRI demonstrated severe bilateral hippocampal atrophy with T2/FLAIR hyperintensity and bilateral cerebellar hemispheric atrophy (Figure 1a). Electroencephalogram (EEG) showed diffuse and bilateral rhythmic theta discharges lasting between 10 and 15 s and without clinical correlate. This pattern was deemed consistent with subclinical rhythmic electrographic discharge of adults (SREDA), a finding of uncertain clinical significance (Figure 1b); a subsequent Video‐EEG recording confirmed absence of clinical manifestations. Repeat CSF analysis was normal (Cell count 0, CSF proteins 16 mg/dL, serum proteins 3532 mg/dL, CSF/Serum protein ratio 0.4%, IgG index 0.4), including within limits neurodegeneration markers. CSF oligoclonal immunoglobulin G bands (IgG‐OCBs) analysis was also executed, showing a Type IV mirror pattern.  At this stage, neuronal antibody testing was requested. Both serum and CSF were tested with a commercial fixed cell‐based assay (CBA) including NMDAR, GABABR, AMPAR, LGI1, and CASPR2, IgLON5, as well as a line blot including Hu, Ri, Sox1, Ma2, GAD, Zic4, and Yo, which tested negative, while an in‐house tissue‐based assay (TBA) performed on rat whole‐brain slices revealed strong neuropil staining in hippocampus and cerebellum (Figure 1c).

**FIGURE 1:**
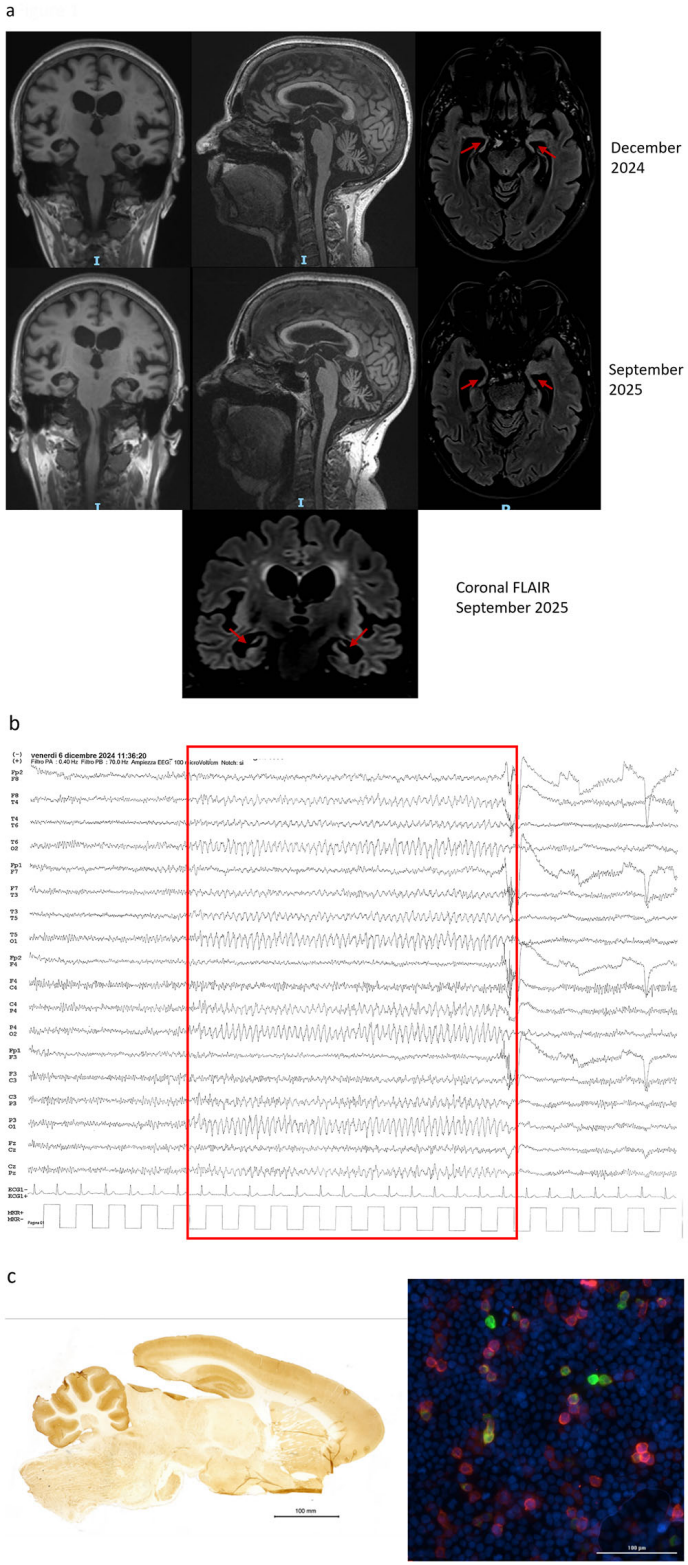
Imaging and laboratory features of our case. (A) Brain MRI findings in December 2024 and September 2025: coronal T1 images show progressive bilateral mesial temporal atrophy (MTA Score 4); sagittal T1 images show vermian and hemispheric cerebellar atrophy; coronal and axial FLAIR images show bilateral hippocampal hyperintensity, more evident on the right side (arrows); contrast has been adjusted to enhance visualization of medial temporal lobe hyperintensity. Comparison between these two timepoints demonstrated no radiological progression. (B) A sequence of monomorphic bilateral theta activity with maximal amplitude over the posterior leads, interrupted by eye opening on command, with no evident behavioral or subjective correlate. This pattern was deemed consistent with SREDA. Filter is set at 0.4–70 Hz, amplitude is 100 microvolt/cm. The whole EEG epoch shown lasts 20 s; the highlighted part is 10.5 s. (C) TBA on rat brain slices with prominent neuropilar staining of cerebellum, cortex, and hippocampus (left image) and live CBA showing SEZ6L2 antibody positivity in CSF (right image; red: SEZ6L2 expression in transfected cells; green: fluorescence shows the antibody binding; blue: DAPI‐stained nuclei).

The specific antigenic target was identified only more than 1 year later. In February 2026, a live CBA performed on the stored serum and CSF samples collected in December 2024, before immunotherapy, detected SEZ6L2‐antibodies in serum (titer 1:20480) and CSF (titer 1:10), leading to a definite AE diagnosis (Figure 1c).

In February 2025 (almost 30 months after onset), after further progression, the patient received four sessions of plasma exchange (PLEX) and IV steroids (methylprednisolone, 1 g/day for 7 days), followed by 2 months of inpatient intensive physical and cognitive rehabilitation. At the end of rehabilitation (April 2025, 2 months after treatment), gait and postural stability had improved (SARA from 14 to 11, with 2 points improvement in gait, and 1 each in stance and sitting items).

During follow‐up, the patient showed substantial clinical stabilization, with no evidence of underlying malignancy on repeated oncological screening. In September 2025 (7 months after the first immunotherapy), repeat MRI was stable (Figure 1a), neuropsychological testing showed slight improvement in visuo‐spatial and executive function (MMSEc 25, CDT 9.5, FAB 14), and neurological examination was largely unchanged. Nonetheless, patient reported subjective worsening. A second course of methylprednisolone (1 g/day for five days) was administered, followed by an oral prednisone taper starting at 62.5 mg/day and gradually reduced (12.5 mg reduction every 2 weeks until 12.5 mg/daily, then 2.5 mg reduction every week) until a maintenance dose of 5 mg/day, reached approximately 3 months after the start of the taper. Nearly 2 weeks later, examination was only marginally improved (SARA score 10, 1 point improvement in limb ataxia), but patient reported subjective improvement in balance.

At the February 2026 follow‐up (5 months after the second IV course), SARA score remained 10, confirming symptom stabilization; no new neurological deficits had appeared. At last available follow‐up, the patient was still using 5 mg/day of prednisone.

Longitudinal plasma NFL monitoring showed high levels at disease peak and a marked reduction occurring between April and September timepoints, following the first administration of steroids and PLEX. SARA score trend confirmed a mild improvement with symptoms stabilization (Figure 2).

**FIGURE 2:**
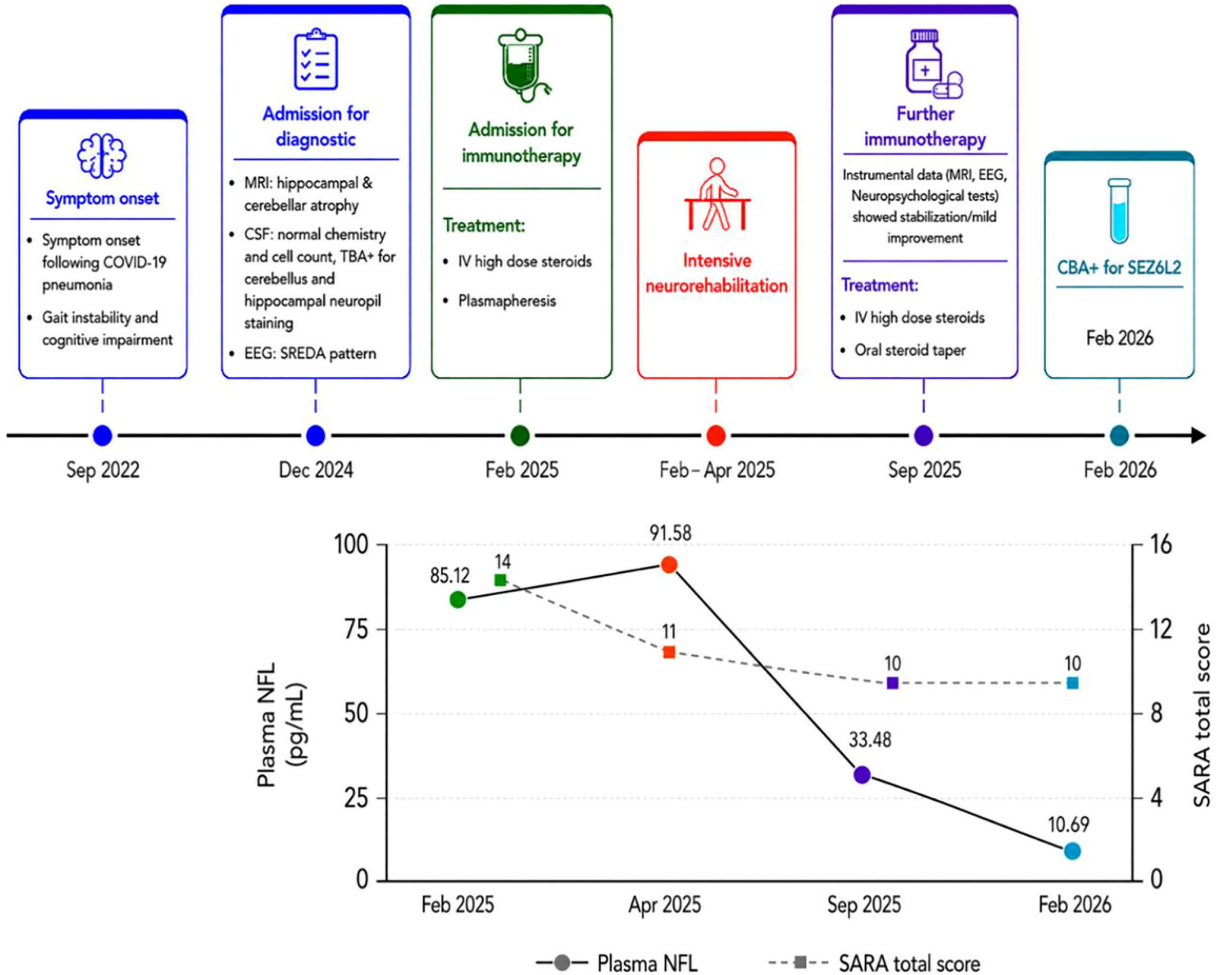
Detailed timeline of patient's history, with relative NFL dosage and scale for the assessment and rating of ataxia (SARA) scores plotted below (produced with assistance of Chat GPT 5, Open AI, 2026).

## 3 Discussion

Our case presented a unique constellation of newly reported features that expand the clinical spectrum of SEZ6L2‐AE, enriching literature on this rare disorder.

First key novel aspects are the clinical features. Whereas previously reported cases of SEZ6L2‐AE have typically described a classic subacute presentation with severe clinical deterioration (Hoshina et al. 2026), in our patient, the disease presented with an insidious and slowly progressive course leading to diffuse cortical and cerebellar atrophy and hippocampal T2 hyperintensities, mimicking a neurodegenerative disease. This is indeed the first report of a slowly progressive evolution in SEZ6L2‐AE.

Notably, despite no clear MRI or CSF clues pointing toward an autoimmune etiology, neuronal antibody screening was still requested, but commonly available commercial panels tested negative. SEZ6L2 antibodies were identified exclusively with an in‐house TBA and live CBA. Clinicians should be aware that an increasing number of antibody‐mediated diseases can mimic neurodegenerative processes and that, in such cases, referring the sample for second‐level in‐house testing is crucial to identify rare, albeit relevant, antibodies.

On the other hand, understanding the beneficial effects of immunotherapy, especially when facing a case with slowly progressive course, can be challenging. In our patient, high‐dose steroids and PLEX led to clinical stabilization. Importantly, NFL showed a reduction over 7 months after the administration of immunotherapy, even though it is impossible at present to determine a cause‐effect relationship. NFL has shown promising results as a prognostic and therapeutic response marker in AE (Mariotto et al. 2019). Our case suggests that longitudinal NFL monitoring could be a particularly useful tool to monitor disease activity, especially in chronic progressive diseases, as demonstrated for IgLON5 disease (Farina et al. 2025). To our knowledge, this is the first SEZ6L2‐AE case showing such a clear NFL decrement.

An important limitation worth mentioning of our report is that brain MRI images from the early phase of the disease (2023) were not available for review, and the corresponding findings could only be derived from the written radiological report; therefore, we could not establish when hippocampal signal changes or temporal/cerebellar atrophy first appeared.

In conclusion, SEZ6L2‐AE should be considered in the differential diagnosis of atypical neurodegenerative processes. Immunotherapy may help limit disease progression, and plasma NFL may be useful for monitoring disease activity.

## Author Contributions

**Carlo Fazio**: conceptualization, investigation, writing – original draft, visualization. **Simone Regalbuto**: investigation, writing – review and editing. **Sebastiano Arceri**: investigation, writing – review and editing. **Davide Comolli**: writing – review and editing. **Alessandra Calculli**: writing – review and editing. **Giulio Trobbiani**: investigation. **Giuseppe Battaglia**: investigation, visualization. **Federica Zuliani**: investigation. **Matteo Gastaldi**: conceptualization, investigation, writing – review and editing, supervision. **Antonio Pisani**: conceptualization, investigation, writing – review and editing, supervision.

## Funding

This work was supported by the Italian Ministry of Health “Ricerca Corrente 2025–2027” granted to IRCCS Mondino Foundation.

## Ethics Statement

The authors confirm that approval from an institutional review board was not required for this work. Informed patient consent was regularly obtained. Both oral and written consent were obtained. We confirm that we have read the journal's position on issues involved in ethical publication and affirm that this work is consistent with those guidelines.

## Conflicts of Interest

The authors declare no conflicts of interest.

## Data Availability

The data that support the findings of this study are available on request from the corresponding author. The data are not publicly available due to privacy or ethical restrictions.
